# Nerve Injury Related to Firearm Extremity Trauma

**DOI:** 10.1177/22925503251392593

**Published:** 2025-11-20

**Authors:** Emma Avery, Harry Lau, Stephanie Stefaniuk, Christine B. Novak, Jana Dengler

**Affiliations:** 1 Division of Plastic, Reconstructive and Aesthetic Surgery, Department of Surgery, 12366 University of Toronto, Toronto, ON, Canada; 2 School of Medicine, 8797 University College Dublin, Dublin, Ireland; 3 Division of Plastic and Reconstructive Surgery, Tory Trauma Program, 12366 Sunnybrook Health Sciences Centre, Toronto, ON, Canada

**Keywords:** firearm trauma, nerve injury, nerve transection, retrospective review, evaluation, traumatisme par balle, lésion nerveuse, dissection nerveuse, analyse rétrospective, évaluation

## Abstract

**Introduction:** Firearm-related extremity trauma with nerve injury can lead to life-altering impairment and disability. This study evaluated the frequency of nerve injury in firearm-related extremity injuries at a Level 1 trauma centre, and the rate of nerve transection in firearm-related peripheral nerve injuries (PNIs) and brachial plexus injuries (BPIs). **Methods:** Following Ethics Board approval, institutional trauma and emergency databases (from 2000 to 2020) were used to identify adults with firearm-related PNI or BPI treated at a Level 1 trauma center. Each case of nerve injury was verified by chart review and excluded isolated digital nerve and other cutaneous nerve injuries. Medical charts were reviewed to retrieve patient and injury data. **Results:** In total, 1957 patients were identified with firearm injuries; the nerve injury study sample included 86 patients (95% males) and 98 nerves injured. The most common upper extremity nerve injured was the radial and/or posterior interosseous nerve (*n* = 30, 25%) and in the lower extremity, the sciatic nerve (*n* = 15, 13%). Nerve transection was confirmed in 21% of cases by surgical exploration (*n* = 19) or ultrasound imaging (*n* = 2). Axonotmetic injuries were confirmed in 20% of cases and in total only 41% of patients had full spontaneous functional recovery. Compared to neurapraxia, neurotmesis injuries had a significantly increased likelihood of concomitant vascular injury (*P* = .007) but not skeletal injuries (*P* = .65). Injury severity score was not associated with nerve injury severity (*P* = .27). **Conclusion:** Nerve transections due to firearm-related trauma occur more frequently than previously believed. Early identification and surgical management of nerve transection injuries is imperative.

## Résumé

**Introduction :** Les traumatismes par balle des extrémités, responsables d'une lésion nerveuse, peuvent être responsables d'atteintes et d'incapacités qui transforment la vie. La présente étude évaluait la fréquence des lésions nerveuses en cas de lésions par balle des extrémités dans un centre de traumatologie de niveau 1 et le taux de dissections nerveuses des lésions des nerfs périphériques (LNP) et des lésions du plexus brachial (LPB) causées par balle. **Méthodologie :** Après avoir obtenu l'approbation du comité d'éthique, les chercheurs ont utilisé les bases de données des traumatismes et de l'urgence de leur établissement (de 2000 à 2020) pour détecter les adultes ayant subi une LNP ou une LPB par balle qui ont été soignés dans un centre de traumatologie de niveau 1. Ils ont analysé chaque cas de lésion nerveuse au dossier et exclu les lésions des nerfs digitaux isolés et les autres lésions des nerfs cutanés. Ils ont examiné les dossiers médicaux pour en tirer les données sur les patients et les lésions. **Résultats :** Au total, 1 957 patients étaient atteints d'une lésion par balle, 86 avaient une lésion nerveuse (95 % de sexe masculin) et 98 nerfs étaient atteints. Le nerf des extrémités supérieures le plus atteint était le nerf radial ou interosseux postérieur (n=30, 25 %) et celui des extrémités inférieures, le nerf sciatique (n=15, 13 %). La dissection nerveuse a été confirmée dans 21 % des cas par exploration chirurgicale (n=19) ou par échographie (n=2). Une axonotmésis a été confirmée dans 20 % des cas, et un total de 40 patients a profité d'un rétablissement fonctionnel complet. Par rapport à la neurapraxie, les neurotmésis étaient beaucoup plus susceptibles d'être associées à une lésion vasculaire concomitante (p=0,007), mais pas à des lésions squelettiques (p=0,65). Le score de gravité des lésions n'était pas associé à la gravité des lésions nerveuses (p=0,27). **Conclusion :** Les dissections nerveuses attribuables à un traumatisme par balle sont plus fréquentes qu'on le croyait. Il est impératif de détecter les lésions par dissection nerveuse et d'en assurer la prise en charge chirurgicale rapidement.

## Introduction

Firearm-related extremity trauma with nerve injury can lead to life-altering impairment and disability. The majority of these injuries occur in young, healthy men.^1–5^ Early and appropriate management of these nerve injuries is essential to maximize functional outcomes. Firearm-related nerve injuries have traditionally been classified as blunt injuries and the management for these injuries is “watchful waiting” with serial clinical and electrodiagnostic (EDX) reassessment.^6^ However, nerve transection secondary to firearm trauma does occur and requires operative management.^4^ Failure to identify nerve transection injuries can lead to delayed surgical management and suboptimal or lack of functional recovery.

Currently, nerve transection in the setting of firearm injuries is identified in the acute injury period if there is an associated vascular and/or skeletal injury that requires surgical management thus exposing the injured nerves. For nerve injuries not requiring operative bony or vascular repair, early diagnosis of the extent of nerve damage is difficult. Magnetic resonance imaging (MRI) is contraindicated in firearm injuries due to the presence of metal shrapnel in the wound. Ultrasound has not traditionally been used for imaging of peripheral nerves in these settings due to lack of technology and expertise.^7,8^ Electromyography (EMG) is not helpful in the acute injury period, as Wallerian degeneration has not yet occurred.^9,10^ Therefore, patients are typically seen in follow-up 6 to 12 weeks after the injury for initial EMG to assess nerve function, and every few months thereafter to determine if spontaneous recovery will occur.

It is well-established that earlier reconstruction of higher-grade nerve injuries leads to superior functional outcomes. However, early operative intervention must be balanced against the likelihood of spontaneous recovery in lower grade nerve injuries.^10^

The purpose of this study was to evaluate the frequency of nerve injury in firearm-related extremity injuries at a Level 1 trauma centre, and to identify the rate of nerve transection in firearm-related peripheral nerve injuries (PNIs) and brachial plexus injuries (BPIs). The secondary aim was to assess the clinical factors associated with higher-grade nerve injuries related to firearm trauma.

## Material and Methods

This retrospective, single-centre study was approved by our institutional Research Ethics Board. The study included adults (18 years and older) with firearm-related upper or lower extremity PNI or BPI who presented to a Level 1 trauma centre between January 1, 2000, and January 31, 2020. Relevant International Classification of Diseases (ICD) ICD-9 and ICD-10 codes (Supplement 1) were used to identify these patients using the institutional trauma and emergency databases.

Identification of a nerve injury was verified by chart review. If a nerve injury was not confirmed by documentation via clinical exam, radiologic imaging or EDX testing, the patient was excluded from the study. Isolated digital nerve and other cutaneous nerve injuries were also excluded. Patient medical charts were reviewed, and data collection included: age at time of injury, gender (binary scale), injury severity score (ISS), ICD 9 or ICD-10 code, concomitant injuries (including presence of associated vascular and skeletal injuries), nerve(s) injured, location of injury, time from injury to diagnosis of nerve injury, duration of hospitalization, diagnostic tests or imaging performed (if applicable), and type of operative or nonoperative treatment.

Nerve injuries were categorized as neurapraxia, axonotmesis or neurotmesis based on clinical assessment. Nerve injuries that had full or near full functional recovery within 3 months of injury were categorized as a neurapraxia. Nerves that were found to be in-continuity via intraoperative assessment, EMG, or imaging, and did not recover within 3 months were categorized as axonotmesis. Nerve injuries that were found to have 1 or more nerve fascicle transected were categorized as a neurotmetic injury. This was confirmed either via direct visualization during intraoperative assessment or via imaging. Within neurotmetic injuries, we classified nerve injuries as partial neurotmetic or complete neurotmetic. Partial neurotmetic injuries were those in which 1 or more nerve fascicles were found divided, but at least 1 nerve fascicle was left intact. Complete neurotmetic injuries were those in which all nerve fascicles had been completely transected. Nerve injuries that did not recover within 3 months and where nerve continuity could not be confirmed were categorized as “unspecified injuries.” Patients who were lost to follow-up prior to 3 months postinjury, and who were not already diagnosed with a neurotmetic injury, were excluded from the study. Clinical assessment at 3 months postinjury differentiated a neurapraxic injury from an axonotmetic injury, in those patients whose affected nerves were found to be in continuity. To assess the natural history of each GSW-related nerve injury in our cohort, we noted the patient's functional recovery at their last follow-up visit (if they were treated with conservative management), or at their final preoperative appointment (if they underwent surgical intervention). Patients were categorized as “fully or nearly-fully recovered” if their Medical Research Council grade of muscle strength (MRC) at final follow-up or preop was 4 or 5. They were considered partially recovery if their MRC was 2 or 3, and nonrecovered if their MRC was 0 or 1.

Concomitant vascular injuries were defined as any blood vessel injury adjacent to the affected nerve that required surgical repair or intervention. Concomitant skeletal injuries were defined as any fractures to bones adjacent to the affected nerve caused by the firearm injury.

Continuous variables were summarized as the mean ± standard deviation (SD) or median and interquartile range (IQR), and categorical variables as frequencies and percentages. Statistical analyses using Chi-square assessed relationships among the categorical variables; Kruskal-Wallis tests were used for all comparisons. A *P-*value < .05 was considered statistically significant.

## Results

**Figure 1.**
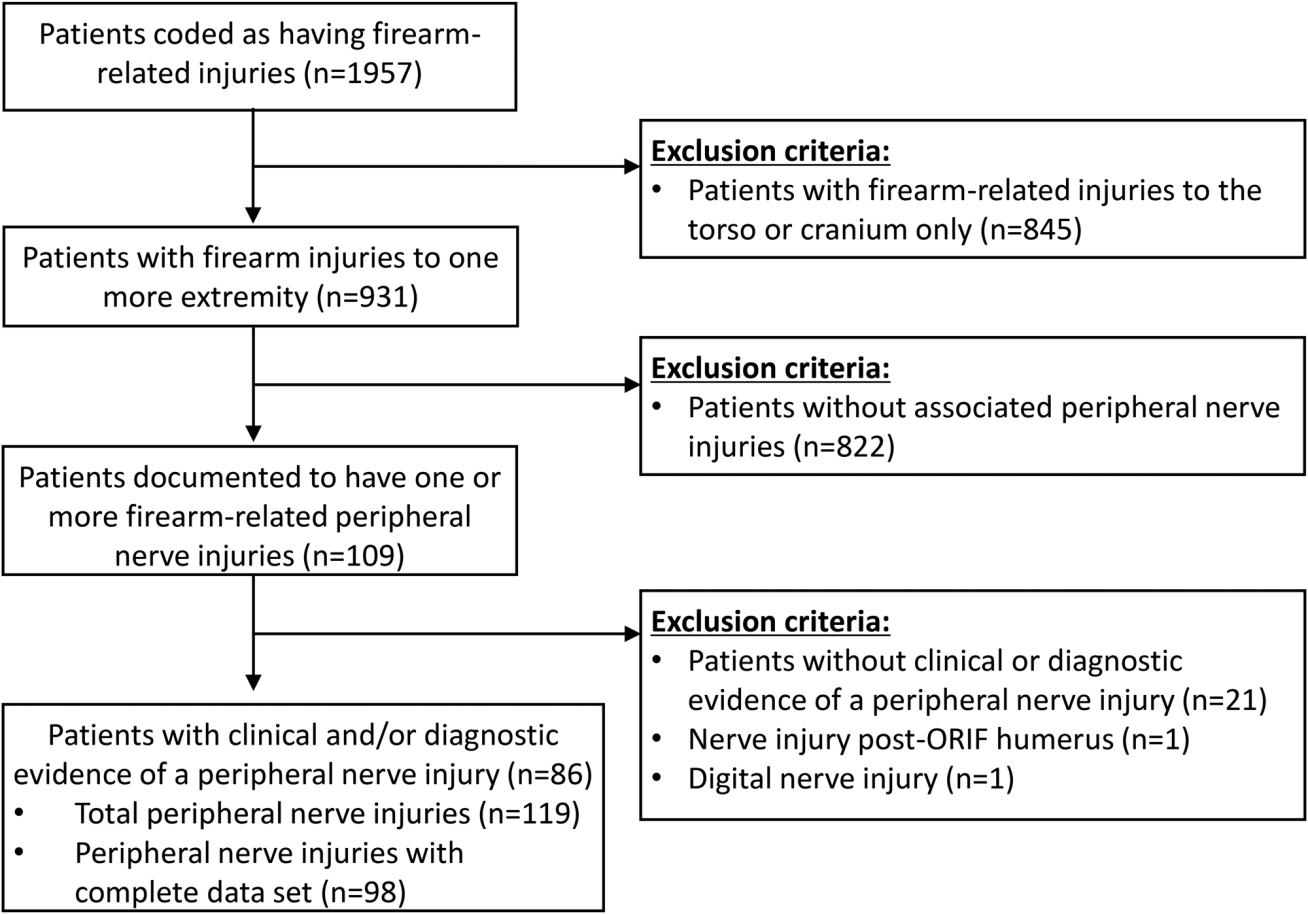
Flow chart demonstrating patient sample selection with inclusion and exclusion criteria.

A total of 1957 patients with firearm injuries presented to our Level 1 trauma centre between 2000 and 2020 (Figure 1). In the database, there were 931 patients (48%) coded with an extremity firearm injury, and 109 patients were coded to have an associated nerve injury in the upper or lower extremity. After chart review, 23 patients were excluded (nerve injury not confirmed *n* = 21; digital nerve injury in the phalanx *n* = 1; nerve injury not associated with firearm *n* = 1) and the study sample included 86 patients (9%) with an extremity firearm injury (median age 25 years, IQR 21-33; 82 males, 95%) with a total of 119 nerves injured in these patients (Table 1). Functional outcomes data were incomplete for 21 nerve injuries and these cases were categorized as “unknown” (Table 2); 98 nerves injuries were included in the final outcomes analysis. The median time from admission to hospital to diagnosis of a nerve injury was 0 days (IQR 0-1 days) and the median duration hospital stay was 9 days (IQR 3-15 days).

**Table 1.** Demographic characteristics.^a^

Age—Years, Median [IQR]	25 [21-33]
Male sex—*n* (%)	82 (95)
Patients injured—*n* (%)	86 (100)
Limbs injured—*n* (%)	87 (100)
Upper limbs injured—*n* (%)	62 (71)
Lower limbs injured—*n* (%)	24 (29)
Nerves injured—*n* (%)	119 (100)
Time from injury to diagnosis of the presence of a nerve injury—day, median [IQR]	0 [0-1]
Duration of hospital stay—day, median [IQR]	9 [3-5]
Duration of follow-up—day, median [IQR]	202 [86-399]

^a^ IQR, interquartile range

**Table 2.** Peripheral nerve injury and associated injuries categorized by severity of nerve injury.^a^

	Neurapraxia *n* (%)	Axonotmesis *n* (%)	Neurotmesis *n* (%)	Unspecified Injury *n* (%)	Unknown *n* (%)	Total nerve injuries *n* (%)
Upper extremity						
Radial/PIN	10 (8)	7 (6)	4 (3)	4 (3)	5 (4)	30 (25)
Ulnar	14 (12)	3 (3)	6 (5)	1 (1)	4 (3)	28 (24)
Median	4 (3)	7 (6)	7 (6)	1 (1)	2 (2)	21 (18)
Brachial plexus	1 (1)	1 (1)	1 (1)	3 (3)	4 (3)	10 (8)
Musculocutaneous	1 (1)	0 (0)	0 (0)	1 (1)	0 (0)	2 (2)
Total UE	30 (25)	18 (15)	18 (15)	10 (8)	15 (13)	91 (76)
Lower extremity						
Sciatic	4 (3)	0 (0)	0 (0)	6 (5)	5 (4)	15 (13)
Peroneal	2 (2)	2 (2)	0 (0)	1 (1)	1 (1)	6 (5)
Femoral	1 (1)	0 (0)	1 (1)	1 (1)	1 (1)	4 (3)
Tibial	1 (1)	0 (0)	1 (1)	0 (0)	0 (0)	2 (2)
Calcaneal	0 (0)	0 (0)	1 (1)	0 (0)	0 (0)	1 (1)
Total LE	8 (7)	2 (2)	3 (3)	8 (7)	7 (6)	28 (24)
Total	38 (32)	20 (17)	21 (18)	18 (15)	21 (18)	119 (100)
Associated Injuries						
Vascular Injury	29*	50	71*	37		187
Skeletal Injury	39**	45	33**	37		154

^a^ Categorical variables expressed as *n* (%). PIN, posterior interosseus nerve; UE, upper extremity; LE, lower extremity

“Unspecified injury” are those injuries that did not recover within 3 months but where nerve continuity could not be confirmed.

“Unknown” are those injuries with no functional outcomes data available.

*P = 0.007; **P = 0.65

The most common nerve injured was the radial and/or posterior interosseous nerve (*n* = 30, 25%) (Table 2). Other upper extremity nerve injuries included ulnar (*n* = 28, 24%), median (*n* = 21, 18%), brachial plexus (*n* = 10, 8%), and musculocutaneous (*n* = 2, 2%). Lower extremity nerve injuries included sciatic (*n* = 15, 13%), peroneal (*n* = 6, 5%), femoral (*n* = 4, 3%), tibial (*n* = 2, 2%), and calcaneal (*n* = 1, 1%). In the analysis of the included 98 nerve injuries, nerve transection (neurotmesis) was seen in 21% of cases (*n* = 21) and this was confirmed by surgical exploration (*n* = 19) and/or high-resolution ultrasound imaging (*n* = 2) (Table 2). Of the confirmed neurotmetic injuries, *n* = 10 were partial neurotmetic, and *n* = 11 were complete neurotmetic injuries. Of those with neurotmetic injuries, 9 patients (43%) were discovered to have nerve transections within 24 h of their GSW, via intraoperative exploration. Six patients (29%) were discovered between day 1 and day 7 postinjury via intraoperative assessment. One patient (5%) was found to have a nerve transection on postop day 1, via ultrasound imaging. Five patients (24%) were discovered to have nerve transections between 2 and 5 months postinjury. Diagnosis of their neurotmetic injury occurred during their reconstructive procedure, without prior knowledge that the patient had suffered a nerve transection. Nine patients were discovered to have a neurotmetic injuries within 24 h There were 20% of nerve injuries that were confirmed axonotmetic injuries. Only 39% of nerve injuries (*n* = 38) recovered spontaneously. The remaining 19% (*n* = 18) did not fully recover within 3 months and were categorized as unspecified injuries as the presence of nerve continuity or transection was not known.

A concomitant vascular injury was seen in 39% (*n* = 46) of patients, and a concomitant skeletal injury was seen in 37% (*n* = 44) of patients. Twenty patients (17%) had both vascular and skeletal injuries, of which 7 (35%) had a neurotmetic injury. Fifty patients (42%) had either a vascular or skeletal injury, of which 15 (30%) had an associated neurotmetic injury. Lastly, 50 patients (42%) had neither a vascular nor skeletal injury, of which 4 (8%) had a neurotmetic injury. Compared to neurapraxia injuries, neurotmetic injuries had a significantly increased likelihood of concomitant vascular injury (*P* = .007), but not skeletal injuries (*P* = .65) (Table 2). ISS was not associated with severity of nerve injury (*P* = .27) (Table 3).

**Table 3.** Severity of nerve injury compared to patient ISS.^a^

Injury Grade	ICD-10 (2005)	ICD-9 (90)
Neurapraxia	17 [8-20]	10 [9-21]
Axonotmesis	22 [21-27]	13 [13-13]
Neurotmesis	10 [10-22]	10 [9-11]
Unspecified injury	17 [17-34]	9 [6-21]
*P*-value	.272	.628

^a^ Continuous variables expressed as median [IQR].

IQR, interquartile range; ISS, injury severity score; ICD, International Classification of Diseases.

“Unspecified injury” are those injuries that did not recover within 3 months but where nerve continuity could not be confirmed.

Prior to surgical intervention, or at last follow-up (if the patient was treated nonoperatively), 41% of the firearm-related nerve injuries had full or near full recovery (ie, MRC 4 or 5), 21% had partial recovery (MRC 2 or 3), and 38% had little to no recovery (ie, MRC 0-1). (Table 4).

**Table 4.** Degree of nerve recovery stratified by severity of nerve injury—prior to surgical intervention, or at last follow-up if no surgical intervention was undertaken.

	Functional Outcomes		
Nerve Injury Severity	No Recovery	Partial Recovery	Full Recovery
Neurapraxia—*n* (%)	0 (0)	0 (0)	38 (100)
Axonotmesis—*n* (%)	4 (20)	16 (80)	0 (0)
Neurotmesis—*n* (%)	21 (100)	0 (0)	0 (0)
Unspecified injury—*n* (%)	12 (63)	5 (26)	2 (11)
Total—*n* (%)	37 (38)	21 (21)	40 (41)

This table does not include the “unknown” injuries with no functional outcomes data for follow-up.

A total of 29% (*n* = 28) of firearm-related nerve injuries underwent operative management, including nerve repair (*n* = 14), nerve grafting (*n* = 8), neurolysis (*n* = 8), tendon transfer (*n* = 7), nerve transfer (*n* = 2), and amputation (*n* = 1).

## Discussion

Our study demonstrated that the occurrence of nerve transection in patients with firearm injuries was not uncommon. Early identification and surgical management of nerve transection injuries in the acute period is essential as delayed surgical management can lead to suboptimal functional recovery. Studies have demonstrated that delayed operative intervention results in less successful muscle reinnervation than those performed at earlier time points following injury.^11–13^

In our study, firearm-related nerve injuries occurred most frequently in the upper extremities of young males, similar to reports in previous studies.^1,3,4^ The radial and ulnar nerves in the upper extremity were the most frequently injured supporting previous studies with similar findings.^3,4,14^ In contrast, other studies have reported the median nerve to be the most frequently injured in the upper extremity.^1^^15–17^ For the lower extremity, the sciatic nerve was most frequently injured in our study with similar findings reported in previous studies of lower extremity gunshot injuries.^1,17^

Historically, firearm-related nerve injuries were believed to cause a concussive type injury to the nerve with indirect trauma related to the temporary cavitation or pressure wave created by the projectile, thermal injury, and compression secondary to edema or subacute scar formation, rather than nerve transection from the direct path of the bullet.^3,17^ Our study found a high frequency of nerve transection injuries which is similar to previous studies reporting nerve transections ranging from 8% to 65%.^3,4,14^^18–23^ In our study, nerve transection (either partial of complete neurotmesis) occurred in 21% of cases, and 20% in-continuity lesions (axonotmesis). There were 39% of patients who had full or nearly full recovery within 3 months following injury. In the remaining 19% of patients who did not recover fully within the first 3 months, the presence of an in-continuity lesion or a nerve transection could not be determined. However, most of these patients (*n* = 12) did not have any functional recovery and therefore may have sustained a partial or complete nerve transection.

Compared to neurapraxic injuries, severe axonotmetic and neurotmetic injuries do not recover without surgical intervention. In our study, in those nerve injuries with functional outcome data, only 41% of nerve injuries had full spontaneous recovery and 21% had partial recovery. Functional data was collected prior to surgical intervention at last follow-up if no surgical invention undertaken. Our group is continuing to collect data on functional recovery following nerve reconstruction. Kim et al reported recovery after gunshot wound nerve injuries in only 12% of patients, and most were surgically treated.^21^ Omer et al reported spontaneous recovery in 69% of patients with gunshot wound nerve injuries between 3 and 9 months after injury, whereas the other 31% showed no recovery.^24^ Luce and Griffin reported spontaneous recovery in 45% after a minimum of 3 months following injury.^18^ Veltre et al reported a full spontaneous recovery rate of 20% to 30% of gunshot wound nerve injuries associated with forearm fractures.^25^ Therefore, patients with in-continuity firearm-related nerve injuries should be closely monitored for recovery as timely surgical intervention may be required.

Skeletal injuries have been frequently associated with firearm-related nerve injuries with a reported range of 27%-70%.^1,3,4,14,18^ Vascular-associated injuries have been associated less frequently with gunshot related nerve injuries and range from 6% to 28%.^1,4,14,26^ In our study, we found a high frequency of concomitant vascular (39%) and skeletal (37%) injuries in patients with firearm-related nerve injuries. In our study, we found that patients who had both vascular and skeletal injuries and patients who had either a vascular or skeletal injury, both had approximately a 1 in 3 chance in having an associated neurotmetic injury. Whereas patients who had neither a vascular nor skeletal injury had a 1 in 12 chance of having an associated neurotmetic injury.

The specific firearm caliber or velocity may affect the occurrence of concomitant vascular or skeletal injuries.^27,28^ While we attempted to collect data on the type of firearm, as well as the firearm caliber or velocity we found that this data was very infrequently reported and analysis of this data was not possible.

Since patients with nerve injuries are not routinely taken to the operating room for early exploration, nonoperative assessment tools are needed to evaluate nerve injuries during the acute phase to avoid missing the opportunity for early intervention when needed. While MRI provides high-contrast resolution in soft tissues and multiplanar images, it is often contraindicated in firearm-related injuries due to remaining metal fragments. Evaluation with EDX is often used to assess neural function. However, in traumatic injuries EDX studies are typically performed 4 to 6 weeks following injury to identify Wallerian degeneration and injury severity. Therefore, EDX studies are not useful to identify transected nerves in the acute phase. Additionally, EDX studies cannot precisely identify the anatomic delineation or underlying cause of a nerve injury. Gousheh reported that although EMG assessments suggested complete nerve transections, surgical exploration revealed the integrity of the nerve if the nerve was in-continuity and compressed by surrounding fibrosis.^29^ Ultrasound is a useful tool in the early diagnosis of nerve transection injuries.^8,30^ Nwawka et al found ultrasound to be useful in patients with firearm-related brachial plexus trauma and attributed this to the higher soft tissue resolution and dynamic imaging of ultrasound.^8^ In an upper extremity cadaveric study, the median, ulnar or radial nerves were transected and high-resolution ultrasound identified nerve transections with 89% sensitivity and 95% specificity.^30^ While ultrasound is helpful in the diagnosis of nerve injury, the quality of imaging and accurate interpretation is dependent upon the technique and experience of the examiner and the available technology.

**Figure 2.**
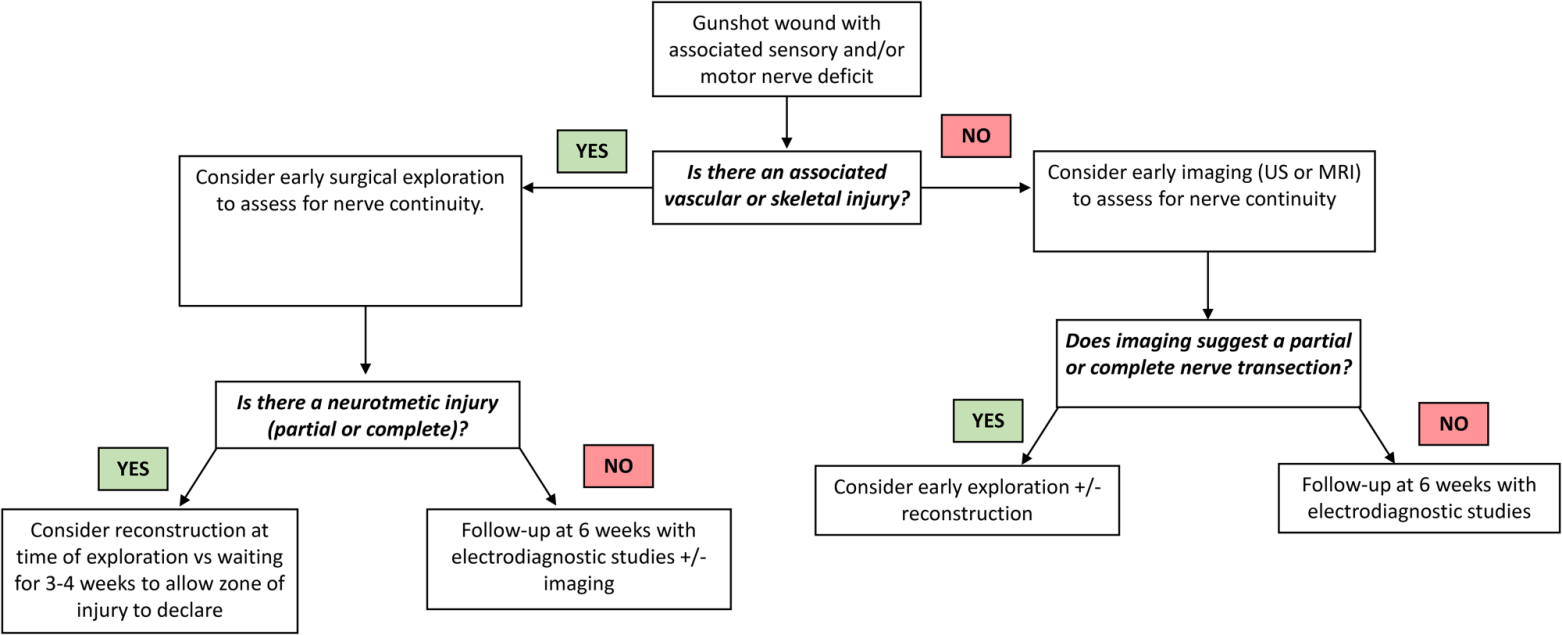
Management algorithm for treating firearm-related peripheral nerve injuries.

The historical approach of “watchful waiting” is based on the concept that in patients with gunshot injuries the nerves typically remain in-continuity and are not transected. Therefore, management has been nonoperative, consisting mainly of observation for 2 to 5 months. However, observation can lead to delayed surgical exploration and the identification of nerve transection. Functional recovery declines with increased time of denervation and after 6 months of denervation time, substantial degeneration of the motor endplates has occurred.^13,31^ Thus, when required, timely surgical repair and reconstruction is necessary to improve functional outcomes.^13^ The authors of this study recommend the following management algorithm for patients with firearm-related peripheral nerve injuries (Figure 2). If a patient presents with a firearm-related peripheral nerve injury, and has an associated vascular or skeletal injury, there should be a high suspicion of a neurotmetic injury (partial or complete). If the patient is going to the operating room for surgical management of their vascular or skeletal injuries, the nerve surgeon may consider nerve exploration at the time of vascular repair or bony fixation. If the patient does have a neurotmetic injury, the nerve surgeon must decide whether to perform nerve reconstruction at the time of injury, or to wait several weeks for the zone of injury to declare itself, ie, plan for nerve repair or reconstruction at 2 to 3 weeks. The disadvantage of immediate nerve reconstruction is difficulty in accurately assessing the zone of injury^32,33^; however, in our experience, the disadvantage of waiting is increased scar burden making dissection more difficult, and nerve retraction in complete transections. If the patient does not have an associated vascular or skeletal injury, then the nerve surgeon must decide whether early exploration is warranted. We recommend utilizing ultrasound or MRI imaging (when possible) to assist in the diagnosis of a neurotmetic or partial neurotmetic injury. If a neurotmetic injury is not diagnosed at the time of injury, we recommend clinical reassessment in 4 to 6 weeks with electrodiagnostic studies and/or imaging.

Limitations of this study include the retrospective study design. There were incomplete functional outcomes data on a subset of patients (*n* = 21) which were not included in the analysis. Additionally, if an injury was not coded as a firearm injury in the database, the patient would not have been included in the study and this may have underestimated the sample with nerve injuries. Information on the type of firearms and bullets which caused the injuries was not available, but presumed to be low-velocity given the urban setting of the trauma centre. This study reviewed injuries at a single Level 1 trauma centre and may not be generalizable to other populations. Future studies are needed to provide additional evidence of our findings.

## Conclusions

Nerve injuries due to firearm-related trauma can cause life-altering impairment and disability. Our study demonstrated that the occurrence of nerve transections is high. Early identification and surgical management of nerve transection injuries is imperative. In cases not undergoing early surgical exploration for other injuries, high-resolution ultrasound is a useful diagnostic tool to assess nerve continuity. Prompt and accurate diagnosis allows early reconstruction, thereby maximizing patient functional outcomes.

## Supplemental Material

sj-docx-1-psg-10.1177_22925503251392593 - Supplemental material for Nerve Injury Related to Firearm Extremity Trauma Supplemental material, sj-docx-1-psg-10.1177_22925503251392593 for Nerve Injury Related to Firearm Extremity Trauma by Emma Avery, Harry Lau, Stephanie Stefaniuk, Christine B. Novak and Jana Dengler in Plastic Surgery
