# Supplementary material for: Nerve Injury Related to Firearm Extremity Trauma
Source: Plast Surg (Oakv). 2025 Nov 20:22925503251392593. Online ahead of print. doi: 10.1177/22925503251392593 (PMC12634383; doi:10.1177/22925503251392593)
Supplement: sj-docx-1-psg-10.1177_22925503251392593 - Supplemental material for Nerve Injury Related to Firearm Extremity Trauma [file sj-docx-1-psg-10.1177_22925503251392593.docx]

| **Supplement 1.** ICD-9 and ICD-10 codes used to select patients | |
| --- | --- |
| **ICD-9** |  |
| GSW injuries | E922.0, E922.1, E922.2, E922.3, E922.8, E922.9, E955.0, E955.1, E955.2, E955.3, E955.4, E955.9, E965.0, E965.1, E965.2, E965.3, E965.4, E970, E985.0, E985.1, E985.2, E985.3, E985.4. |
| Limb injuries | 812.0, 812.1, 812.11, 812.3, 812.13, 812.19, 812.20, 812.21, 812.30, 812.31, 812.40, 812.41, 812.42, 812.43, 812.44, 812.49, 812.50, 812.51, 812.52, 812.53, 812.59, 812.9, 813.0, 813.1, 813.2, 813.3, 813.4, 813.5, 813.6, 813.7, 813.8, 813.10, 813.11, 813.12, 813.13, 813.14, 813.15, 813.16, 813.17, 813.18, 813.21, 813.22, 813.23, 813.31, 813.32, 813.33, 813.40, 813.41, 813.42, 813.44, 813.50, 813.51, 813.52, 813.54, 813.80, 813.90, 814.0, 814.1, 814.2, 814.3, 814.4, 814.5, 814.6, 814.7, 814.8, 814.9, 814.1, 814.12, 814.13, 814.14, 814.15, 814.16, 814.17, 814.18, 814.19, 815.0, 815.10, 815.1, 815.11, 815.2, 815.12, 815.3, 815.13, 815.4, 815.14, 815.9, 815.19, 816.0, 816.10, 816.1, 816.11, 816.2, 816.12, 816.3, 816.13, 817.0, 817.1, 818.0, 818.1, 819.0, 819.1, 820.0, 820.1, 820.3, 820.9, 820.10, 820.11, 820.13, 820.19, 820.20, 820.21, 820.22, 820.30, 820.31, 820.32, 821.0, 821.10, 821.1, 821.11, 821.20, 821.21, 821.22, 823.23, 821.30, 821.31, 821.32, 821.33, 822.01, 823.10, 823.20, 823.30, 823.81, 823.91, 824.0, 824.1, 824.2, 824.3, 824.4, 824.5, 824.6, 824.7, 824.8, 824.9, 825.0, 825.1, 825.2, 825.21, 825.22, 825.23, 825.24, 825.25, 825.30, 825.31, 825.32, 825.33, 825.34, 825.35, 826.0, 826.1, 827.0, 827.1, 828.0, 828.1, 832.0, 832.10, 832.1, 832.11, 832.12, 832.13, 832.14, 832.19, 832.2, 832.3, 832.4, 832.9, 835.0, 835.10, 835.1, 835.11, 835.2, 835.12, 835.3, 835.13, 833.0, 833.1, 833.2, 833.3, 833.4, 833.5, 833.9, 833.11, 833.12, 833.13, 833.14, 833.15, 833.19, 834.0, 834.10, 834.1, 834.11, 834.12, 834.2, 836.0, 836.1, 836.3, 836.4, 836.50, 836.51, 836.52, 836.53, 836.54, 836.59, 836.60, 836.61, 836.62, 836.63, 836.64, 836.69, 837.0, 837.1, 838.2, 838.12, 838.3, 838.13, 838.15, 838.16, 838.5, 838.6, 839.8, 841.0, 841.1, 841.22, 841.3, 841.8, 841.9, 842.0, 842.1, 842.10, 842.11, 842.12, 842.13, 842.19, 842.2, 842.9, 843.0, 843.1, 843.8, 843.9, 844.0, 844.1, 844.2, 844.8, 844.9, 845.1, 845.10, 845.12, 845.13, 845.2, 845.3, 845.9, 880.20, 880.23, 880.29, 880.13, 880.3, 880.19, 880.9, 881.0, 881.10, 881.1, 881.11, 881.2, 881.12, 881.20, 881.20, 881.22, 882.2, 883.0, 883.1, 883.2, 884.0, 884.1, 885.0, 886.0 887.0, 887.2, 887.4, 890.0, 890.1, 890.2, 891.0, 891.1, 891.2, 892.0, 892.1, 893.0, 893.1, 893.2, 894.0, 894.1, 895.0, 903.1, 903.2, 903.3, 903.4, 903.5, 903.8, 903.9, 904.0, 904.2, 904.3, 904.4, 904.52, 904.6, 904.7, 904.8, 912.8, 913.8, 913.9, 916.8, 916.9, 919.8, 923.0, 923.11, 923.3, 923.20, 924.0, 924.10, 924.20, 924.3, 927.1, 927.2, 927.3, 928.1, 928.21, 928.11, 928.3 |
| Nerve injuries | 953.4, 954.0, 955.0, 955.1, 955.2, 955.3, 955.4, 955.5, 955.6, 955.7, 955.8, 955.9, 956.0, 956.1, 956.2, 956.3, 956.4, 956.5, 956.8, 956.9, 957.0. |
| **ICD-10** |  |
| GSW injuries | W31.0, W32.1, W33.0, W33.1, W34.0, W34.1, X72, X73.0, X73.1, X73.2, X73.8, X73.9, X74.0, X74.8, X74.9, X93, X94.0, X94.1, X94.2, X94.8, X94.9, X95.0, X95.8, X95.9, Y22, Y23.0, Y23.1, Y23.2, Y23.3, Y23.8, Y23.9, Y24.0, Y24.8, Y24.9, Y35. |
| Limb injuries | S40, S41, S42, S45, S47, S50, S51, S52, S53, S58, S61, S62, S63, S68, S70, S71, S72, S73, S75, S81, S82, S83, S91, S92, S93, S98, |
| Nerve injuries | S14.3, S44.0, S44.1, S44.2, S44.3, S44.4, S44.5, S44.8, S44.9, S54.0, S54.1, S54.2, S54.3, S54.8, S54.9, S64.0, S64.1 , S64.2, S64.3, S64.4, S64.8, S64.9, S74.0, S74.1, S74.2, S74.8 S74.9, S84.0, , S84.1, S84.2, S84.8, S84.9, S94.0, S94.1, S94.2, S94.3, S94.8, S94.9. |
| ICD, International Classification of Diseases | |
